# Administration of Levetiracetam in Traumatic Brain Injury: Is it Warranted?

**DOI:** 10.7759/cureus.9117

**Published:** 2020-07-10

**Authors:** Jason DJohn, Ramzi Ibrahim, Prasanna Patel, Kaitlyn DeHoff, Nina Kolbe

**Affiliations:** 1 General Surgery, St. Joseph Mercy Oakland, Pontiac, USA; 2 Internal Medicine, St. Joseph Mercy Oakland, Pontiac, USA; 3 Medicine, St. Joseph Mercy Oakland, Pontiac, USA; 4 Surgery, St. Joseph Mercy Oakland, Pontiac, USA

**Keywords:** tbi, levetiracetam, seizure, gcs, keppra

## Abstract

Background

Antiepileptic agents are recommended to prevent early post-traumatic seizures (PTS) within seven days of injury in patients with severe traumatic brain injury (TBI). These agents are not routinely recommended for patients with mild-to-moderate TBI, defined as Glasgow Coma Scale (GCS) score > 8. At St. Joseph Mercy Oakland, levetiracetam (LEV) is commonly prescribed to prevent PTS. The objective of this study was to evaluate the appropriateness of LEV use in patients with mild, moderate, and severe TBI.

Methods

This retrospective cohort study evaluated the use of LEV in adult patients admitted with TBI over a five-year period. Patients who were younger than 18 years, had a history of seizures, were transferred to a tertiary center, or succumbed to their injuries were excluded. The primary outcome was appropriateness of LEV use. Secondary outcomes included duration of LEV treatment and rate of seizures.

Results

Of the 448 patients evaluated, 36 patients were excluded. Of the 412 included patients, 403 (97.8%) had a non-severe TBI, defined as GCS score > 8. In patients with non-severe TBI, 153 (38%) received LEV and 94 (23.3%) received LEV for more than seven days. Additionally, 105 (26.1%) patients with non-severe TBI were discharged with a prescription for LEV despite not having a seizure during hospitalization. All six patients with non-severe TBI who experienced a seizure were receiving LEV.

Conclusions

Inappropriate use of LEV is common in patients admitted with non-severe TBI, with many patients continuing LEV post-discharge. With careful patient selection, patients with mild and moderate TBI likely do not need seizure prophylaxis with LEV. Education on appropriate indication and duration of LEV in patients with TBI is warranted.

## Introduction

Traumatic brain injury (TBI) is a leading cause of long-term neurological disability [1]. During the post-TBI period, the increased risk of seizures plays an integral role as a source of further impairment and is highly associated with long-term unfavorable outcomes, as well as a predictor of future development of epilepsy [2]. The risk of early post-TBI seizures in the first seven-day period ranges from 4% to 25% [3]. This risk is seen in patients with primarily a severe TBI, identified as a Glasgow Coma Scale (GCS) score of ≤8. These patients typically present with subdural hematoma, epidural hematoma, intracranial hematoma, linear/depressed fracture, or cortical contusion [4]. Mitigating such risks with a seven-day period of anti-seizure medications is of high priority in the post-TBI period, as recommended by the Brain Trauma Foundation [5].

Levetiracetam (LEV) is extensively used as a broad-spectrum antiepileptic medication for a wide array of seizure types, including juvenile myoclonic epilepsy and idiopathic generalized epilepsy. Its efficacy was compared favorably with statistical data on other antiepileptic medications such as topiramate, lamotrigine, and vigabatrin [6]. It has become a standard practice largely due to its assumed safer profile, without the need for monitoring of serum concentrations and its minimal drug interactions, especially when compared with phenytoin, another drug historically used for anti-seizure purposes [7]. These presumptions that favor LEV over phenytoin have been inconsistent in the literature, and it is recommended that LEV be used conservatively. However, the practice of administering LEV as prophylaxis for patients with a mild-to-moderate TBI goes against the conservative use and has therefore been analyzed at our institution.

We sought to evaluate the utility of administering LEV in mild-to-moderate TBIs, defined as a GCS score > 8. We also wanted to determine if LEV was being prescribed appropriately according to the national TBI guidelines (seven-day duration with no reported seizure activity). With recent literature suggesting a more conservative use of LEV in TBI, our goal is to decrease the unnecessary administration of LEV in an effort to prevent possible adverse medication reactions as well as to decrease hospital costs.

## Materials and methods

This is a retrospective chart review evaluating the use of LEV in TBI patients at St. Joseph Mercy Oakland Hospital (a community hospital in Pontiac, Michigan) over a five-year period. Data retrieval began after the St. Joseph Mercy Oakland Hospital Institutional Review Board approved the study. Between the years 2015 and 2019, patients with mild, moderate, and severe TBI who were admitted for either a subdural, epidural, or subarachnoid hemorrhage were included in the study. Patients who were younger than 18 years, had a history of seizures, had chronic use of anti-seizure medications, were transferred to a tertiary center, or succumbed to their injuries were excluded from the study. Data were gathered by an extensive chart review including GCS score, administration of LEV, duration of LEV treatment, and development of seizures. The primary outcome was appropriateness of LEV use in regard to the severity of TBI. Secondary outcomes included duration of LEV treatment and the occurrence of seizures. The statistical tests used to analyze the data were Fisher's exact test and Mann-Whitney U test. These outcomes were reviewed and results were compared. A p-value of <0.05 (95% confidence interval) was used to assess the significance of seizure occurrence.

## Results

**Figure 1:**
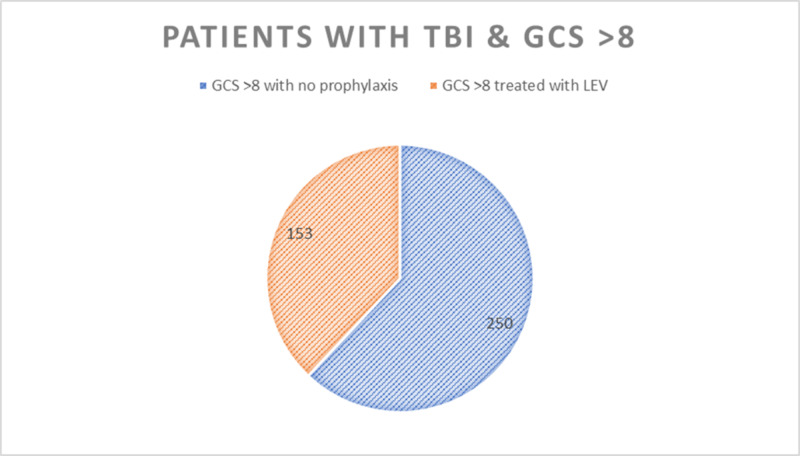
In total, 403 patients in the study had a GCS score > 8, out of which 153 patients were treated with LEV. TBI, traumatic brain injury; GCS, Glasgow Coma Scale; LEV, levetiracetam

**Figure 2:**
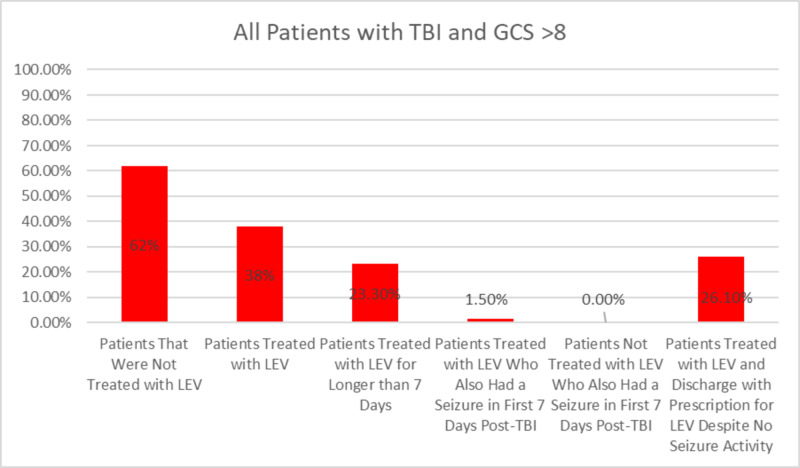
This chart highlights the percentages of patients that fall under the group of a TBI with GCS score > 8. Out of all of the patients who had GCS score > 8 (403 patients), 38% of them were treated with LEV, 23% were treated for longer than seven days, 1.5% had seizure activity (who also were treated with LEV), and 26% were treated with LEV and prescribed additional LEV upon discharge despite no seizure activity. None of the patients who were not treated with LEV encountered a seizure. TBI, traumatic brain injury; GCS, Glasgow Coma Scale; LEV, levetiracetam

A total of 448 patients were reviewed during the years 2015 to 2019, with 36 patients being excluded. Fisher's exact test and Mann-Whitney U test were used to analyze the data. Out of 412 patients, 403 had a GCS score of >8 (97.8%). In the GCS > 8 group, 153 patients (38%) received LEV and 94 patients (23.3%) received LEV for more than seven days. Using Fisher's exact test, there were six patients (1.5%) in the GCS > 8 group who had a seizure (p<0.005). All six of these patients were being treated with LEV prior to the development of seizures. Additionally, 105 patients (26.1%) of the GCS > 8 group were discharged with a prescription for LEV despite not having a seizure during hospitalization. Figures 1, 2 below illustrate these findings.

## Discussion

The use of LEV has heightened in the recent years [8] due to its decreased drug interactions compared with phenytoin, which has notoriously been used for seizure prophylaxis. These presumed benefits of LEV have been inconsistent in the literature of the past decade. In 2010, a prospective randomized single-blind study with 52 patients compared the use of LEV and phenytoin in the early post-TBI period and concluded that LEV improved long-term outcomes according to the Glasgow Outcome Scale and Disability Rating Scores [9]. However, according to a multicenter prospective study conducted in 2013, both phenytoin and LEV had no significant differences in seizures rates, adverse drug reactions, or mortality [10]. More recently, a retrospective cohort study conducted in 2018 showed a lower incidence of seizures in those treated with LEV versus without prophylaxis, although this difference did not reach statistical significance [11]. Conservative use of LEV is recommended largely due to insufficient evidence.

Over-prescribing this drug poises unnecessary added risks to patients. The use of LEV encompasses potential risks that may cause more harm than benefit in these cases, with adverse effects including fatigue, somnolence, neuropsychiatric dysfunction, and upper respiratory infections [12,13]. Therefore, the purpose of this study was to evaluate the utility of LEV administration and determine if it is being used appropriately, according to national TBI guidelines. We found that 38% of patients who encountered a non-severe TBI were being prescribed LEV. Additionally, 23.3% of patients were given LEV longer than advised by current national guidelines (seven-day treatment) and 26.1% of patients were discharged with a prescription for LEV despite not having a seizure during hospitalization. Interestingly, all 6 patients that were in the GCS > 8 group that had a seizure were being treated with LEV (p<0.005), raising suspicion that a confounding variable was placing these patients at an added seizure risk. There were no documented seizures in patients that were not being treated with LEV.

## Conclusions

With careful patient selection, patients with mild-to-moderate TBI likely do not need seizure prophylaxis with LEV. Conservative use of LEV will decrease not only hospital costs but also the risk of an adverse medication reaction. Furthermore, if LEV prophylaxis is started, the duration of therapy shall not exceed seven days if there was no documented seizure activity during the hospitalization.
